# Survival and Disease Recurrence in Patients with Duodenal Neuroendocrine Tumours—A Single Centre Cohort

**DOI:** 10.3390/cancers13163985

**Published:** 2021-08-06

**Authors:** Oddry Folkestad, Hans H. Wasmuth, Patricia Mjønes, Reidun Fougner, Øyvind Hauso, Reidar Fossmark

**Affiliations:** 1 Department of Gastrointestinal Surgery, St. Olav’s Hospital, Trondheim University Hospital, 7030 Trondheim, Norway; oddfol@siv.no; 2 Department of Gastrointestinal Surgery, Vestfold Hospital, 3103 Tønsberg, Norway; 3 Department of Clinical and Molecular Medicine, Faculty of Medicine and Health Sciences, Norwegian University of Science and Technology (NTNU), 7030 Trondheim, Norway; hans.wasmuth@ntnu.no (H.H.W.); Patricia.mjones@ntnu.no (P.M.); oyvind.hauso@stolav.no (Ø.H.); 4 Department of Pathology, St. Olav’s Hospital, Trondheim University Hospital, 7030 Trondheim, Norway; 5 Department of Radiology, St. Olav’s Hospital, Trondheim University Hospital, 7030 Trondheim, Norway; Reidun.Lyngvi.Fougner@stolav.no; 6 Department of Gastroenterology and Hepatology, St. Olav’s Hospital, Trondheim University Hospital, 7030 Trondheim, Norway

**Keywords:** neuroendocrine tumour, duodenum, endoscopy, surgery, survival, recurrence, outcome

## Abstract

**Simple Summary:**

Neuroendocrine tumours of the upper part of the small intestine are rare. They are slow growing but may spread to lymph nodes or other organs already when the tumours are small. Such tumours may be treated by endoscopic removal or by an operation. In the current study we present the treatment results of 32 patients with this rare tumour. We found that the long-term survival was long, and patients more often died from other diseases. The survival was associated with the growth rate of the tumours and whether all the tumour tissue could be removed. Endoscopic removal was sufficient for smaller tumours <10 mm, whereas a high proportion of tumours 10–20 mm have lymph node metastases that must be removed by an operation to make patients tumour free. None of the tumours that were perceived as cured after removal recurred after an average follow-up time of 4.8 years.

**Abstract:**

Background: Duodenal neuroendocrine tumours (D-NETs) are rare but increasingly diagnosed. This study aimed to assess the overall survival and recurrence rate among patients treated for D-NETs. Methods: Patients with D-NETs were retrospectively reviewed with a median follow-up time of 4.8 years (range 0.0–17.2 years). Results: A total of 32 patients with median age 68.0 years were identified. Fifteen patients underwent surgery while ten patients underwent endoscopic treatment. Mean estimated overall survival for the entire population was 12.1 years (95% CI 9.5–14.7 years), while 5-year overall survival was 81.3%. Tumour grade G1 was associated with longer mean estimated survival compared to G2 tumours (13.2 years versus 4.4 years, *p* = 0.010). None of the 23 patients who underwent presumed radical endoscopic or surgical resection had disease recurrence during follow-up. Tumours <10 mm could be treated endoscopically whereas a high proportion of patients with tumours 10–20 mm should be considered for surgery. Conclusion: Patients with D-NETs had long overall survival, and mortality was more influenced by other diseases. Both endoscopic and surgical resections were effective as no recurrences were diagnosed during follow-up.

## 1. Introduction

Duodenal neuroendocrine tumours (D-NETs) are rare neoplasms that are infrequently found during upper gastrointestinal (GI) endoscopies, some causing symptoms while others are found incidentally. The overall incidence of D-NETs is 0.19/100,000 in the United States [1] and these tumours comprise 2.8% of all GI-NETs [2]. The incidence of D-NETs has shown an increasing trend from 0.027/100,000 in 1983 to 1.1/100,000 in 2010 [3] paralleling the increasing volume of upper GI endoscopies [4]. The median age of presentation is in the 6th decade and there is a slight male predominance (1.5:1) [1]. The majority of D-NETs are found in the duodenal bulb and descending part of the duodenum, while up to 20% are found in the periampullary region [5,6,7,8]. Tumours involving the ampulla of Vater are often considered a separate entity with clinical behaviour more similar to pancreatic neoplasms [6,9,10]. D-NETs may be discovered incidentally in at least 15% to 33% of cases [6,7,11,12,13]. D-NETs are often small, more than 75% are <2 cm in size and localized in the mucosa or submucosa at the time of diagnosis [5,6,11,12]. However, metastases to regional lymph nodes are present in 40–60% of the patients at diagnosis [14,15], whereas liver metastases are seen in <10% of patients at diagnosis [16]. According to the consensus guidelines of the European Neuroendocrine Tumour Society (ENETS) treatment of D-NETs is mainly guided by tumour size, i.e., tumours <1 cm should be treated endoscopically, tumours >2 cm should be considered for surgical resection whereas treatment of tumours 1–2 cm is not standardized [10]. A recent systematic review identified only few studies reporting outcomes and recurrence rates after treatment of D-NETs and the evidence supporting the current treatment recommendations is limited [17,18]. We therefore aimed to assess the overall survival and recurrence rate in a cohort of patients with D-NETs.

## 2. Materials and Methods

### 2.1. Patients

A retrospective analysis of medical records for patients with a histologically confirmed diagnosis of D-NETs was conducted. The analysis was approved by the Norwegian Centre for Research Data and the Regional Committee for Medical and Health Research Ethics, South-East Norway. A total of 34 patients with D-NETs were identified from the archives of Department of Pathology at St Olav’s hospital by searching the SNOMED codes T64xxx (small intestinal tumour) and M824xx (NET) in the time period between 1 January 1998 and 31 August 2020. Patients with SNOMED codes T64xxx and M824xx who had jejunoileal NETs were excluded. Two patients with duodenal neuroendocrine carcinoma (NEC), which is a different tumour biological entity, were excluded from further analyses. Data on patient demographics, symptoms at diagnosis attributable to D-NET, diagnostic procedures related to D-NETs and the presence of synchronous cancers were extracted from the medical records. The pathology reports were reviewed for the following tumour variables: number, location, size, depth of invasion, resection margin status, immunohistochemical features, presence or absence of vascular and lymphatic invasion, lymph node metastases. Follow-up data were obtained from the medical records.

### 2.2. Radiological Imaging at Diagnosis

Pre-treatment abdominal computed tomography (CT), somatostatin-receptor scintigraphy scan, ^68^Ga-DOTATOC positron emission tomography (PET) from 2019 and endoscopic ultrasonography (EUS) reports were reviewed, and the presence, size and location of presumed lymph node metastases, liver metastases and extra-abdominal metastases at baseline were recorded.

### 2.3. Surgical Resection, Endoscopic Treatment or Diagnostic Biopsy

Patients underwent either surgical treatment with local resection (LR) or pancreaticoduodenectomy (PD), endoscopic treatment (ET) with formal endoscopic mucosal resection (EMR) or removal of small tumours (<5 mm) by endoscopic biopsy forceps. Patients who only underwent diagnostic biopsy without subsequent treatment were classified as diagnostic biopsy (DB). The operations categorized as local resection (LR) were either a duodenotomy with tumour resection, distal gastric resection with partial inclusion of the 1st part of the duodenum or segmental resection of the duodenum. Further treatment during follow-up was recorded, which included pharmacological treatment, surgical removal of metastases and peptide receptor radionuclide treatment (PRRT).

### 2.4. Disease Stage and Severity

Disease stage and severity at the time of diagnosis was classified according to the TNM-classification 8th edition [19] and European Neuroendocrine Tumour Society (ENETS) staging system [20,21] and were based on findings on upper GI endoscopy, endoscopic ultrasonography (EUS), radiological imaging, surgical reports and histopathological examinations. ENETS stage 0–IIIa is termed localized disease, stage IIIb nodal disease and stage IV distant metastatic disease [22]. Proliferation rates were estimated by the Ki-67 index of primary tumours and classified according to the World Health Organization (WHO) grade (G1–3) [23].

### 2.5. Survival and Disease Recurrence

The patients were followed until they were registered as dead, or until the last follow-up visit resulting in a median follow-up time of 4.8 years (0.0–17.2 years). Upper GI endoscopies were performed in patients treated with endoscopic resection at 3–12 months intervals the first year and then adjusted according to tumour characteristics as well as patient age and comorbidity. CT scans were performed at regular intervals (6–12 months) of patients treated with curative intention and surveillance was clinically meaningful, similar to the recommendations in ENETS guidelines that evolved during the observation period. Additional CT scans and somatostatin receptor imaging were performed if disease recurrence was suspected during follow-up or to evaluate treatment effect or disease progression. Deaths were categorized as caused by D-NET based on information found in the medical record. Patients who died due to metastasis or complications from advanced D-NET disease or treatment of the disease, were listed as D-NET related.

### 2.6. Statistical Analyses

Descriptive data are presented as frequency (n (%)) or median (range), as appropriate. Overall survival with comparisons between groups was analysed by Kaplan–Meier with log-rank test. The Fishers exact test was used for comparisons of categorical variables, whereas Mann–Whitney U test or Kruskal–Wallis test were used for comparison of continuous variables between groups. *p*-values < 0.05 were considered statistically significant. All statistical analyses were conducted using IBM SPSS Statistics version 25.0 (IBM Corporation, Armonk, NY, USA).

## 3. Results

### 3.1. Patient and Tumour Characteristics

Thirty-two patients had a histopathological D-NET diagnosis (Table 1). Median age at the time of diagnosis was 67.0 years (29–86 years), while median age at the time of resection was 68.0 years (33–86 years). The symptoms attributable to D-NET at diagnosis were abdominal pain (*n* = 8), non-ulcer dyspepsia (*n* = 5), weight loss (*n* = 4), symptoms due to peptic ulcer disease (*n* = 3), jaundice (*n* = 2), GI-bleeding (*n* = 1) and diarrhoea (*n* = 1). Three patients had functional tumours; all were sporadic gastrinomas with hypergastrinemia (175–17,225 pM) whereof two had liver metastases at diagnosis. The patients with gastrinomas had diarrhoea and/or symptomatic peptic ulcers. Seven patients were asymptomatic at diagnosis. The initial D-NET diagnosis was based on endoscopic biopsies in 29 patients, while three patients were operated on due to other diseases with a D-NET found incidentally in the surgical resection specimen. The median tumour size was 12 mm (2–35 mm). Thirty patients had a single D-NET while two patients had multiple primary tumours. Twenty patients had localized disease, six patients had nodal disease, while five patients had distant metastatic disease. In one patient treated endoscopically the disease stage could not be determined since radiological examinations were not performed. The majority of D-NETs were located in the duodenal bulb (*n* = 17), eight were located in the descending part of the duodenum and seven tumours in the periampullary region (Table 2). Most patients (*n* = 28) had slowly proliferating G1 tumours, while four patients had G2 tumours. The median follow-up time was 4.8 years (0.0–17.2 years). Three patients had synchronous cancer (pancreatic adenocarcinoma, gastric adenocarcinoma and hepatosplenic T-cell lymphoma).

### 3.2. Tumour Size and Disease Stage

Median tumour size was associated with disease stage. In patients with localized disease the median tumour size was 8.5 mm versus 15.5 mm in patients with nodal disease and 17.0 mm in the patients with distant disease (*p* = 0.022). It is of clinical interest that none of ten patients with tumours <10 mm had nodal disease or distant metastases, whereas seven of fifteen patients (46.7%) with tumours 10–20 mm and four of seven patients (57.1%) with tumours >20 mm had nodal disease or distant metastases (Table 3).

### 3.3. Endoscopic Treatment or Diagnostic Biopsy

Seventeen patients underwent endoscopic treatment (ET) (*n* = 10) or diagnostic biopsy (DB) only (*n* = 7). The tumour size in the ET group was median 7.0 mm (5–25 mm), notably one patient had a 25 mm pedunculated polyp treated by radical endoscopic snare resection. Median tumour size in the DB group was 11.5 mm (8–20 mm). All ten patients had G1 tumours in the ET group, while four of seven patients in the DB group had G1 tumours (*p* = 0.127). All patients who underwent ET had localized disease and were considered endoscopically tumour-free after the procedure. The patients who underwent DB only had either advanced disease (Table 1) or significant comorbidity and were by definition not tumour-free during follow-up. There were no deaths during follow-up in the ET group, while four patients died in the DB group: two patients due to D-NET related disease, one patient due to hepatosplenic T-cell lymphoma and one patient due to cerebral infarction. There was no disease recurrence during follow-up in the ET group with a follow-up time of 5.3 years (0.6–17.1 years). Fifteen of 17 patients who underwent ET or DB were followed by upper GI endoscopies and/or CT scans performed once or multiple times during the first three years.

### 3.4. Surgical Treatment

Fifteen patients were treated by surgical resection (Table 1). The median tumour size was 17.0 mm (2–35 mm) and all were G1 tumours. Five patients underwent pancreaticoduodenectomy (PD) and were considered radically resected. Ten patients were treated by local resection (LR) with or without lymphadenectomy. The LR procedures comprised Billroth 2 procedures (*n* = 3), duodenotomy with local excision of the D-NET (*n* = 4) and segmental duodenal resections (*n* = 3). All operations were elective procedures, except for one emergency operation due to massive upper GI bleeding. There were two incidental findings of D-NET in the surgical specimens of patients operated for other diseases: gastric cancer (D-NET size 2 mm) and pancreatic cancer (D-NET size 3 mm). The patients who underwent PD were 62.0 years (37–71 years) and tumour size was 12.0 mm (3–35 mm), while the LR group was 68.0 years (41–86 years) (*p* = 0.426) with tumour size 17.5 mm (2–28 mm) (*p* = 0.624). The presence and number of positive lymph nodes in the surgical specimens are presented in Table 2. As expected, tumour size was associated with increasing T-stage (*p* = 0.004) and increasing T-stage was also associated with positive nodal status (*p* < 0.001). There was no significant association between WHO grade and tumour size, nodal or distant metastasis status. Eight of ten patients in the LR group and all five patients in the PD group were considered radically resected and tumour free after surgery and none relapsed during a median follow-up of 8.0 years (0.01–15.5 years). The follow-up included cross-sectional imaging 6–12 months after surgery and subsequently at one-year intervals for the majority of the patients. None of the fifteen patients who underwent surgery died from NET related disease.

### 3.5. Periampullary Tumours

A subgroup of seven patients (21.9%) had periampullary tumours. Median tumour size was 16.5 mm (8–35 mm) versus 12.0 mm in the non-ampullary group (*p* = 0.195). Six patients had G1 tumours while one patient had a G2 tumour, similar to the distribution in non-ampullary tumours (*p* = 0.874). Four patients were surgically treated; three underwent operation with PD, one was operated with LR. Three patients were treated by EMR. Five of the seven patients were considered tumour free after their procedures. Three patients had local disease confined to the ampulla of Vater, four had nodal metastases while none had distant metastases. The D-NETs had infiltrated beyond the submucosa in five patients. The patients were followed for 8.0 years (2.3–10.0 years). No recurrences were discovered during the follow-up period, while one patient died due to cerebral infarction.

### 3.6. Overall Survival

Nine of the 32 patients died during follow-up. Three patients died due to D-NET related disease, whereof one died perioperatively due to surgical complications and multiorgan dysfunction after an emergency procedure. Four patients died of other cancers (pancreatic cancer, gastric cancer, hepatosplenic T-cell lymphoma and lung cancer), while two patients died of other diseases than cancer. The mean estimated overall survival for all patients was 12.1 years (95% CI 9.5–14.7 years), while the 5-year overall survival was 81.3%. None of the patients who received endoscopic treatment died during a median follow-up of 5.3 years (0.6–17.2 years), whereas the survival of patients who only underwent a diagnostic biopsy was 4.6 years (1.7–7.4 years) and 5-year survival of 57.1%. Mean estimated overall survival for surgically treated patients was 11.6 years (95% CI 8.0–15.2 years) and 5-year overall survival was 80.0% (*p* = 0.869). Tumour grade G1 was associated with longer survival compared to G2, with mean estimated overall survival of 13.2 years (95% CI 10.6–15.8 years) versus 4.4 years (95% CI 0.7–8.2 years) and 5-year overall survival of 85.7% versus 50.0%, respectively (*p* = 0.010). The three patients with gastrinoma were alive after 0.5, 1.4 and 12.3 years of follow-up, respectively. Periampullary location did not seem to affect survival, as these patients had a mean estimated overall survival of 9.0 years (95% CI 0.9–7.3 years) and 5-year survival of 85.7% versus 11.3 years (95% CI 8.2–14.4 years) and 5-year survival of 80.0% in patients with non-ampullary D-NET (*p* = 0.331).

## 4. Discussion

D-NETs are heterogeneous tumours with increasing incidence. Most D-NETs are slowly proliferating low-grade tumours, as found in the present study where 87.5% of the patients had G1 tumours. The ENETS guidelines suggest that treatment of non-ampullary D-NETs should be guided by tumour size. It is recommended that tumours <1 cm or >2 cm in size, should be considered for endoscopic or surgical treatment, respectively, whereas the treatment recommendation for tumours 1 to 2 cm is not standardized [10,24]. We found that tumour size was associated with disease stage, as previously reported by others [5,13,25,26,27]. It is of particular interest to interventional endoscopists that none of the tumours <10 mm had nodal or distant metastases. However, a considerable proportion of patients (46.7%) with tumours 10 to 20 mm had nodal or distant disease, which highlights the need for careful pre-treatment evaluation of intermediate size tumour. Others have found that none of 58 non-functioning D-NETs <1 cm in size had non-localized disease versus 10 of 37 of tumours >1 cm, supporting the safety of using 1 cm as cut-off [27]. However, two other studies have found lymph node metastases in 10–13% of patients with D-NETs <10 mm [5,28]. Previous studies have shown that 40–60% of D-NETs overall have regional metastases at diagnosis [14,15], in the present study 18.8% of the patients had nodal disease and 15.6% had distant metastases at diagnosis. Lymph node metastases were found in 63.6% of the surgically treated patients and nodes with and without metastases were more frequent in surgical specimens after PD than after LR, as reported by Margonis et al. [13]. However, the prognostic significance of positive lymph node status has been questioned, as it does not seem to predict overall survival [13,29,30]. This may be explained by the indolent nature of D-NETs and questions the benefit of lymphadenectomy in patients with high risk [13].

Nine patients died during follow-up, whereof only three died due to D-NET related disease. D-NETs per se had low mortality in the current observational study setting where most patients have been treated with resection with curative intention. In an epidemiological study with propensity score matching resection of D-NETs were associated with longer survival regardless of tumour size [31]. However, randomized trials to compare the outcome of endoscopic or surgical resection with curative intention with surveillance have, given the rarity of D-NETs, not been performed so far. The mean overall survival was 12.1 years and the 5-year survival 81.3%. Furthermore, we found 5-year survival rates for local, nodal and distant disease to be 80.0%, 83.3% and 80.0%, respectively. Similar 5-year overall survival rates of 73.9% to 86.1% have been reported previously [5,13,29,32]. In SEER data the 5-years survival rates for local, regional and distant stages were 68%, 55% and 46% and median survival 107 months, 101 months and 57 months respectively [1]. We found that overall survival was longer in patients with G1 tumours versus G2 tumours, as also found by others [33]. This may be explained by G2 tumours being both more advanced at the time of diagnosis and aggressive during follow-up, in terms of tumour size, lymphovascular invasion, duodenal wall invasion depth as well as presence of metastases [27,32].

None of the 23 patients who were considered tumour-free after endoscopic or surgical treatment had disease recurrence during follow-up. Few others have described long-term results after treatment, but small D-NETs selected for endoscopic resection seem to have a very low risk of later recurrence [24,27,34]. However, little is known about the clinical course of untreated small D-NETs and randomized trials are needed.

Strengths of this study include complete and long-term follow-up of a patient cohort with evaluation of both survival and disease recurrence. Analyses of data derived from large patient registries often lack information about disease recurrence, a factor which is particularly important when evaluating treatment outcome of indolent D-NETs. The study was limited by its retrospective design and that patients were thus not randomized with respect to treatment modality that could affect outcome.

## 5. Conclusions

Patients with D-NETs had a long overall survival, and the mortality was more influenced by other diseases. Both endoscopic and surgical resections were effective as no recurrences were diagnosed during follow-up. Endoscopic treatment of tumours <10 mm seems sufficient, whereas a high proportion of patients with tumours 10–20 mm in size have non-localized disease and patients should be considered for surgical resection.

## Figures and Tables

**Table 1:** Demographics, disease and intervention in patients with D-NETs.

Variable	Surgery		Endoscopy		Total *n* = 32	*p*-Value
	PD *n* = 5	LR *n* = 10	Endoscopic Treatment *n* = 10	Diagnostic Biopsy *n* = 7		
Age at diagnosis, years, median (range)	62.0 (37–70)	64.5 (29–86)	61.5 (33–76)	76.0 (67–81)	67.0 (29–86)	0.031
Age at resection, years, median (range)	62.0 (37–71)	68.0 (41–86)	61.5 (33–76)		68.0 (33–86)	0.035
Male, *n*	2	8	2	4	16	0.063
ENETS stage, *n*						0.014
Localized disease	1	7	10	2	20	0.007
Nodal disease	4	1	0	1	6	0.007
Distant disease	0	2	0	3	5	0.050
NA	0	0	0	1	1	
WHO grade, *n*						0.049
G1	5	10	9	4	28	
G2	0	0	1	3	4	
Tumour free after treatment, *n*	5	8	10	0	23	<0.001
Synchronous cancer, *n*	1	1	0	1	3	0.606
Deaths during follow-up, *n*	1	4	0	4	9	0.059
Deaths due to surgical complications	0	1			1	
30-day mortality	0	1	0	0	1	0.532
90-day mortality	0	1	0	1	2	0.583
Treatment during follow-up, *n*						
SSA	0	2	0	3	5	0.084
Chemotherapy	0	1	0	1	2	0.583
PRRT	0	1	0	1	2	0.583
Interferon	0	1	0	0	1	0.532

PD: pancreaticoduodenectomy; LR: local resection; ENETS: European Neuroendocrine Tumour Society; NA: not available; WHO: World Health Organization; G: grade; SSA: somatostatin analogue; PRRT: peptide receptor radionuclide treatment.

**Table 2:** Tumour and histopathological characteristics of patients with D-NETs.

Variable	Surgery		Endoscopy		Total *n* = 32	*p*-Value
	PD *n* = 5	LR *n* = 10	Endoscopic Treatment *n* = 10	Diagnostic Biopsy *n* = 7		
Location of tumour, *n*						0.183
Duodenal bulb	0	6	7	4	17	
Descending part	2	3	1	2	8	
Periampullary	3	1	2	1	7	
Tumour size, mm, median (range)	12.0 (3–35)	17.5 (2–28)	7.0 (5–25)	11.5 (8–20)	12.0 (2–35)	0.108
Pts. with pos. LN in surgical specimen, *n*	4/5	3/6				
LN resected	130 (2–24)	1 (0–4)			1 (0–24)	0.002
Positive LN	2 (0–7)	0.5 (0–4)			1 (0–7)	0.132
Resection margins, *n*						0.344
R0	5	7	2		14	
R1	0	2	2		4	
R2	0	1	0		1	
NA			6	7	13	

PD: pancreaticoduodenectomy; LR: local resection; NA: not available; Pts: patients; LN: lymph nodes; IHC: immunohistochemistry.

**Table 3:** Tumour size, disease stage and intervention.

Tumour Size				
Variable	<10 mm *n* = 10	10–20 mm *n* = 15	>20 mm *n* = 7	*p*-Value
Disease stage				0.046
Local	10	7	3	
N+	0	4	2	
M+	0	3	2	
NA		1		
Grade				0.957
G1	9	13	6	
G2	1	2	1	
NA	1			
Hormone production, *n*				0.167
Functional	0	3	0	
Non-functional	9	11	6	
NA	1	1	1	
Intervention				0.023
Biopsy only	1	5	1	
Endoscopic resection	7	2	1	
Surgical resection	2	8	5	

NA: not available.

## Data Availability

The original data cannot be made public available.
